# Herbicide tolerance and gene silencing stability over generations in the ricin bio-detoxicated castor bean

**DOI:** 10.1186/s43141-022-00303-w

**Published:** 2022-01-28

**Authors:** Natália L. de Sousa, Glaucia B. Cabral, Francisco J. L. Aragão

**Affiliations:** Embrapa Recursos Genéticos e Biotecnologia, PqEB W5 Norte, Brasília, DF 70770-900 Brazil

**Keywords:** Imazapyr, *Ricinus communis*, Transgenic castor bean

## Abstract

Castor bean (*Ricinus communis* L.) is an important cultivated oilseed. Seeds contain ricinoleic acid, a valuable product for a variety of industries. Castor cake is a residue of ricinoleic manufacture and could be used as animal feed due to its high amount of protein. However, castor cake contains ricin and RCA_120_, both highly toxic and allergenic proteins. In 2017, we reported the development of a transgenic event (named TB14S-5D) with an undetectable amount of ricin/RCA_120._ In the present work, we evaluate TB14S-5D for tolerance to the herbicide imazapyr, as it contains the selectable marker gene, *ahas*, which was previously isolated from *Arabidopsis thaliana* and contains a mutation at position 653 bp. In addition, we demonstrated that the ricin coding genes are stably silenced over three generations.

## Introduction

Castor bean (*Ricinus communis* L.) is an oilseed found worldwide and commonly cultivated in tropical and subtropical regions. India, Mozambique, China, and Brazil are the major producers. India is responsible for about 80% of the world’s production [1]. Castor oil is the most important product, with great value in industry, especially to produce lubricants, medicines, and cosmetics, as it contains high amounts of ricinoleic acid, a viscous and highly stable fatty acid [2].

Castor cake is the by-product generated after oil extraction. It is mainly used as a fertilizer and soil conditioner. However, it could also be used in animal feed because it contains high amounts of protein and essential amino acids*.* Nevertheless, it is extremely toxic due to the presence of the highly toxic/allergenic proteins, ricin and RCA_120_ [3, 4]. Ricin is a highly toxic ribosome-inactivating protein (RIP) present in castor seeds’ endosperm, formed by two chains. Chain B is a lectin that binds to glycoproteins/glycolipids present on the cell surface and that allows ricin to enter animal cells. Chain A inactivates the ribosomes by depurination of one adenosine in the conserved loop on the 28 S rRNA subunit, resulting in cell death [5]. RCA_120_ is a strong hemagglutinin, composed of two A chains and two B chains, highly similar to ricin (90 and 84%, respectively) [3].

The demand for castor products has increased, leading to the need for increased production/yield [6]. Castor crop yield is affected by several factors, such as weed management, which is a challenge, since castor is very sensitive to competition and initially takes time to grow, in contrast to weeds, which grow faster [6]. To control weeds in large crops, several agronomic practices must be taken into account. However, mechanical control is very expensive, and the use of herbicides is more efficient, especially if herbicides with a distinct mode of action are used [6, 7].

We generated an RNA interference-mediated ricin-silenced castor bean event, named TB14S-5D [8]. The vector pRicRNAi used to generate the event contained an intron hairpin cassette to silence ricin and the *gus* gene (coding for a β-glucuronidase) and the mutated *Atahas* gene (coding for an acetohydroxyacid synthase, with a mutation at position 653 bp resulting in a serine to asparagine substitution), which confers tolerance to imidazolinone herbicides [9]. The T_1_ generation showed an undetectable amount of ricin in seeds and no hemagglutination activity [8]. In addition, seed protein extracts were not toxic to both IEC-6 cells and mice. T_1_ progeny also revealed high expression of *gus* and in vitro tolerance to imazapyr. This class of herbicides inhibits the activity of the AHAS enzyme, impairing the biosynthesis of isoleucine, leucine, and valine [10].

This work aims to evaluate the tolerance of the transgenic event TB14S-5D to imidazolinone, as well as the stability of ricin/RCA_120_ silenced, and *gus* expression in subsequent generations.

## Materials and methods

### Test for tolerance to imazapyr

**Fig. 1:**
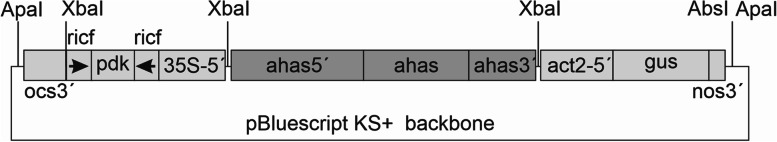
Diagram representing the vector pRicRNAi used to obtain the transgenic castor bean event TB14S-5D. The vector contains the *ahas* gene (ahas5′: *ahas* gene promoter; ahas: *A. thaliana* AHAS coding sequence; ahas3′: *ahas* gene terminator), the intron-hairpin cassette to silence ricin endogenous genes (35S-5′: *35S* promoter of *Cauliflower mosaic virus*; pdk: *Flaveria trinervia pdk* intron; ocs3′: octopine synthase terminator from *Agrobacterium tumefaciens*, ricf: 460-bp ricin gene fragments cloned in sense and antisense orientations) and the *gus* gene (act2-5′: actin 2 promoter from *A. thaliana*; gus: β-glucuronidase coding region; nos3′: nopaline synthase terminator from *A. tumefaciens*)

The transgenic castor bean line (named TB14S-5D) used in this study was previously generated as described by Sousa et al. [8]. It was obtained by embryonic axes bombarded with the vector pRicRNAi (Fig. 1).

Seeds collected from non-transgenic and T_3_ generation of transgenic lines were sown in 5 dm^3^ plastic pots containing fertilized soil. Twenty-one-day-old plantlets were sprayed with the herbicide (imazapyr) (using a solution of 1 g/L) at the final dose of 100 g ha^−1^ and 250 g ha^−1^, observed and photographed after 75 days. Seven transgenic and non-transgenic (wild type; WT) plants were used for each herbicide concentration, evaluated under greenhouse conditions. Experiment was repeated twice.

### Quantification of ricin content

Quantification of ricin content in mature seeds of the transgenic event TB14S-5D [generations T_1_, T_2_ and T_3_ (homozygous)] was carried out using ELISA [11]. The homozygosis was verified by testing 30 plants from the T_3_ generation for the *gus* gene expression. For protein extraction, 200 mg of tissue (endosperm) was ground in liquid nitrogen and vortexed in 600 μL of phosphate-buffered saline (PBS) for 30 min at 4 °C. The mixture was centrifuged at 20,800 *g* for 60 min at 4 °C, and the aqueous phase was collected. Total protein was quantified using the Quick Start Bradford Protein Assay (Bio-Rad Laboratories). For ricin detection, goat antiserum (Santa Cruz Biotechnology) was used, raised against a peptide located at the N-terminus of the ricin precursor. A standard curve was produced using purified ricin A (Sigma, L9514). The limit of detection was determined as 80 pg/μg total protein in the 50 μL well. Absorbance was measured in a microplate reader (Bio-Rad) at 405 nm.

### Hemagglutination assay

Hemagglutination assay was carried out in a 96-well microtiter plate [8]. Total proteins from endosperm of transgenic and non-transgenic seeds were extracted as previously described. Each well contained 50 μL phosphate-buffered saline (PBS) and 50 μL of RCA_120_ (initial concentration of 0.1 μg/μL), 50 μL total proteins isolated from transgenic and non-transgenic castor bean endosperm serially diluted by a ratio of ½ starting with 2 μg total protein/μL. The blank was made with 50 μL PBS. Fifty microliters of a 2% suspension (diluted in 0.15 M NaCl) of cow (*Bos indicus*) red blood cells were added to each well and gently mixed. Plates were incubated at room temperature for 2 h, and results were recorded. The titer was expressed as the reciprocal of dilution factor of the last well showing hemagglutination activity. Samples were observed using an inverted microscope.

### Beta-glucuronidase (GUS) histochemical assay

Leaf tissues were analyzed for in situ localization of GUS activity [12].

### Statistical analysis

Data were analyzed by analysis of variance (ANOVA) at *p* < 0.01 followed by Dunnett’s test to compare between treatments as implemented in GraphPad Prism 6.0 software.

## Results and discussion

**Fig. 2:**
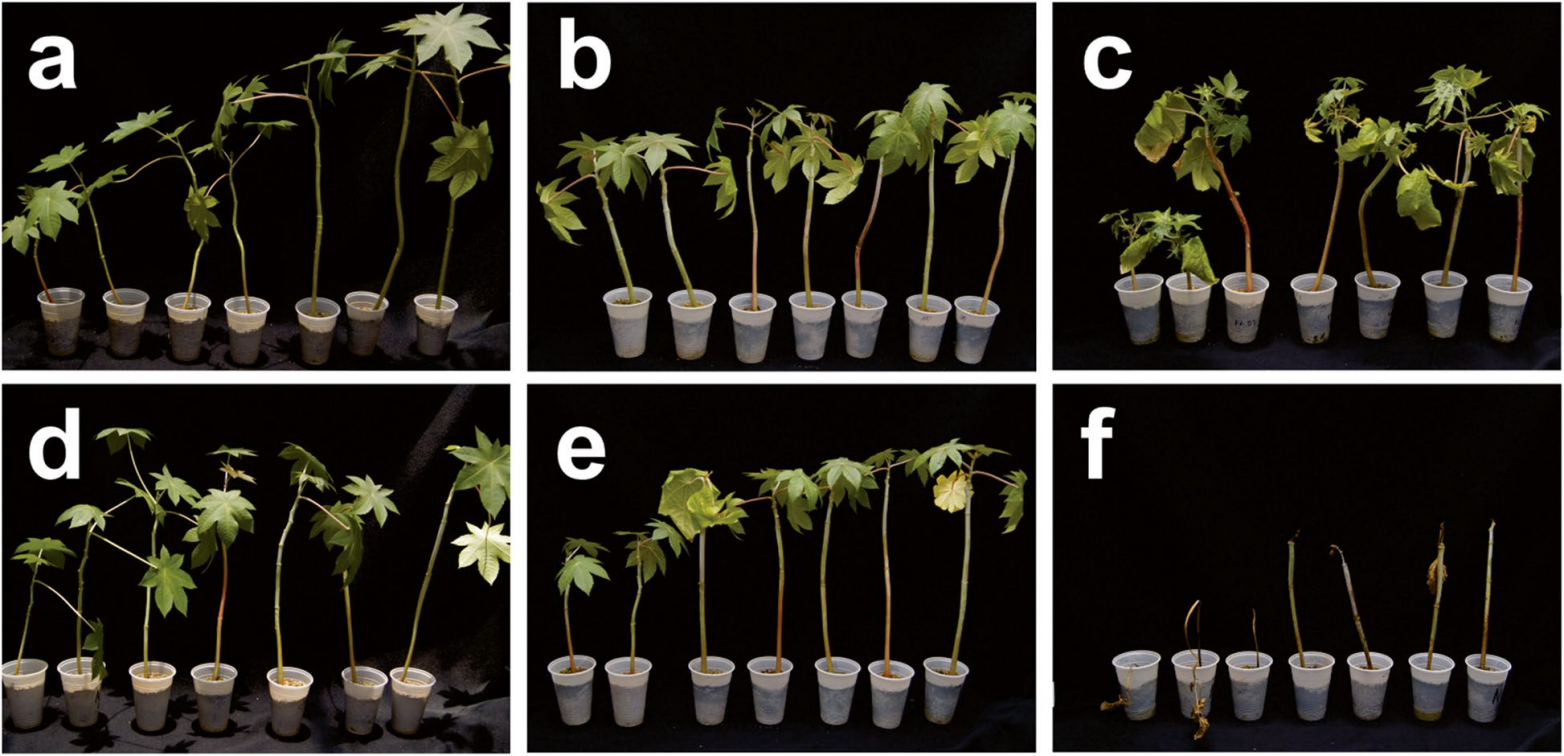
Evaluation of transgenic castor bean event TB14S-5D at 75 days post application of herbicide. Twenty-one day-old plantlets from the transgenic (**a**–**c**) and non-transgenic (**d**–**f**) lines. Plantlets were sprayed with 0 g ha^−1^ (**a** and **d**), 100 g ha^−1^ (**b** and **e**), 250 g ha^−1^ (**c** and **f**) imazapyr

We reported here the tolerance of transgenic castor bean to herbicidal molecule imazapyr (imidazolinone) using a mutated *Atahas* gene from *A. thaliana*. Transgenic plants treated with 100 g/ha of imazapyr presented no symptoms of intoxication (Fig. 2b). In contrast, WT plants treated with the same concentration started to present multiple shoots in the apical meristem (Fig. 2e). At 250 g/ha of Imazapyr, non-transgenic plants presented symptoms of red vein and death of apical meristems (Fig. 2f). In contrast, the transgenic line presented tolerance up to 250 g/ha of Imazapyr, showing only signs of multiple shoots in the apical meristem (Fig. 2c). All WT plants treated with 250 g/ha of Imazapyr died after 75 days, and the transgenic plants remained healthy, and normally growing (Fig. 2f, c), similar to the transgenic and non-transgenic plants with no herbicide applied (Fig. 2a, d). There is a considerable interest in generating herbicide tolerant castor bean varieties. Imidazolinone tolerance was achieved by both conventional and molecular breeding in rice, soybean, sugar beet, cowpea, and sugarcane [9, 13–17]. The event TB14S-5D showed high tolerance to 250 g ha^−1^ imazapyr, which is 3.5-fold the commercial recommended dose for weed control. Additionally, we have previously demonstrated that this line presented a Mendelian segregation in the F_1_ generation [8]. It makes the transgene easier to transfer to other genotypes. Although event TB14S-5D has yet to be tested under field conditions, there is the prospect that its cultivation can be used as an efficient tool as part of a weed control strategy.

**Fig. 3:**
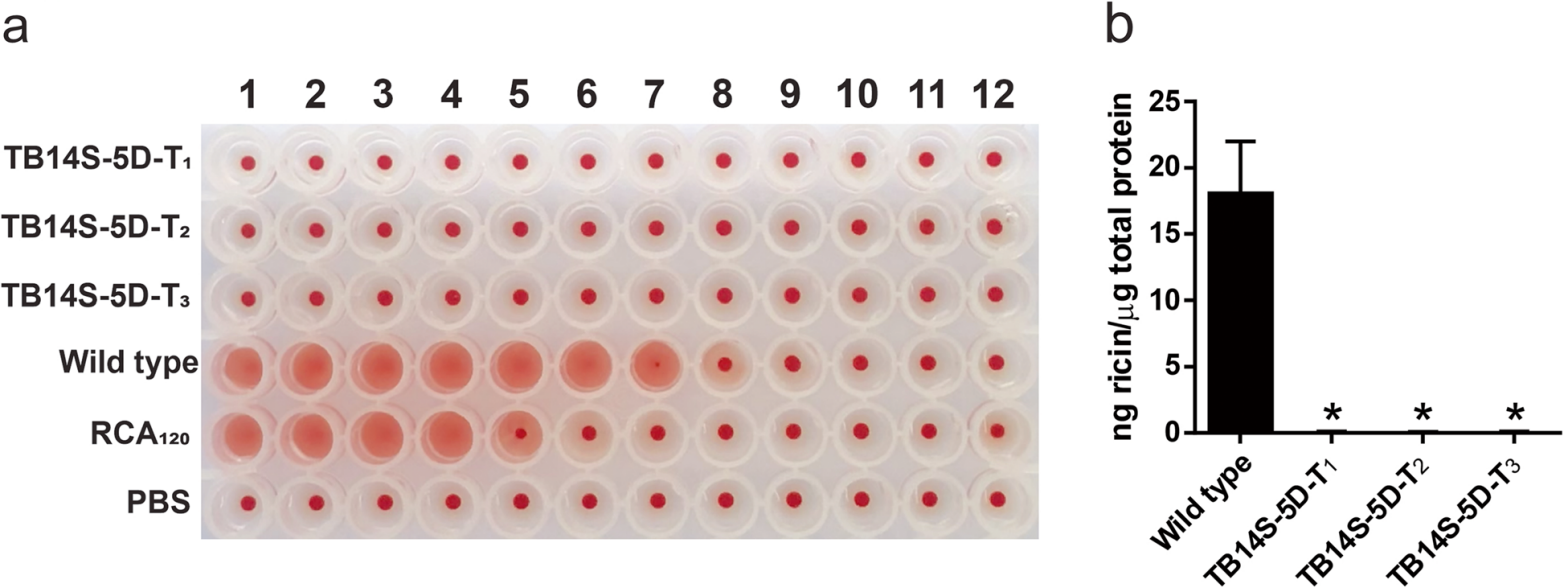
Ricin/RCA_120_ proteins are not detected in seeds from three generations of transgenic bio-detoxified event TB14S-5D. **a** Proteins from transgenic [TB14S-5D, generations T_1_, T_2_, and T_3_ (homozygous)] and non-transgenic (wild type) seeds were tested for their capacity to hemagglutinate red blood cells (2% suspension). Protein concentration was serially diluted by a ratio of ½ from rows 1 to 12, starting with 2 μg total protein/μL. RCA_120_ (starting with 0.1 μg/μL) was used as a positive control and PBS was a negative control. Agglutinated RBC formed a diffuse mat, whereas non-agglutinated RBC sediment formed a dot at the bottom of the well. **b** ELISA was used to detect and quantify ricin in the endosperm. Ricin was detected in non-transgenic seeds (wild type seeds) and could not be detected in positive transgenic seeds from generations T_1_, T_2_, and T_3_. Asterisks represent significant differences compared to control (*P* < 0.01, *n* = 9)

**Fig. 4:**
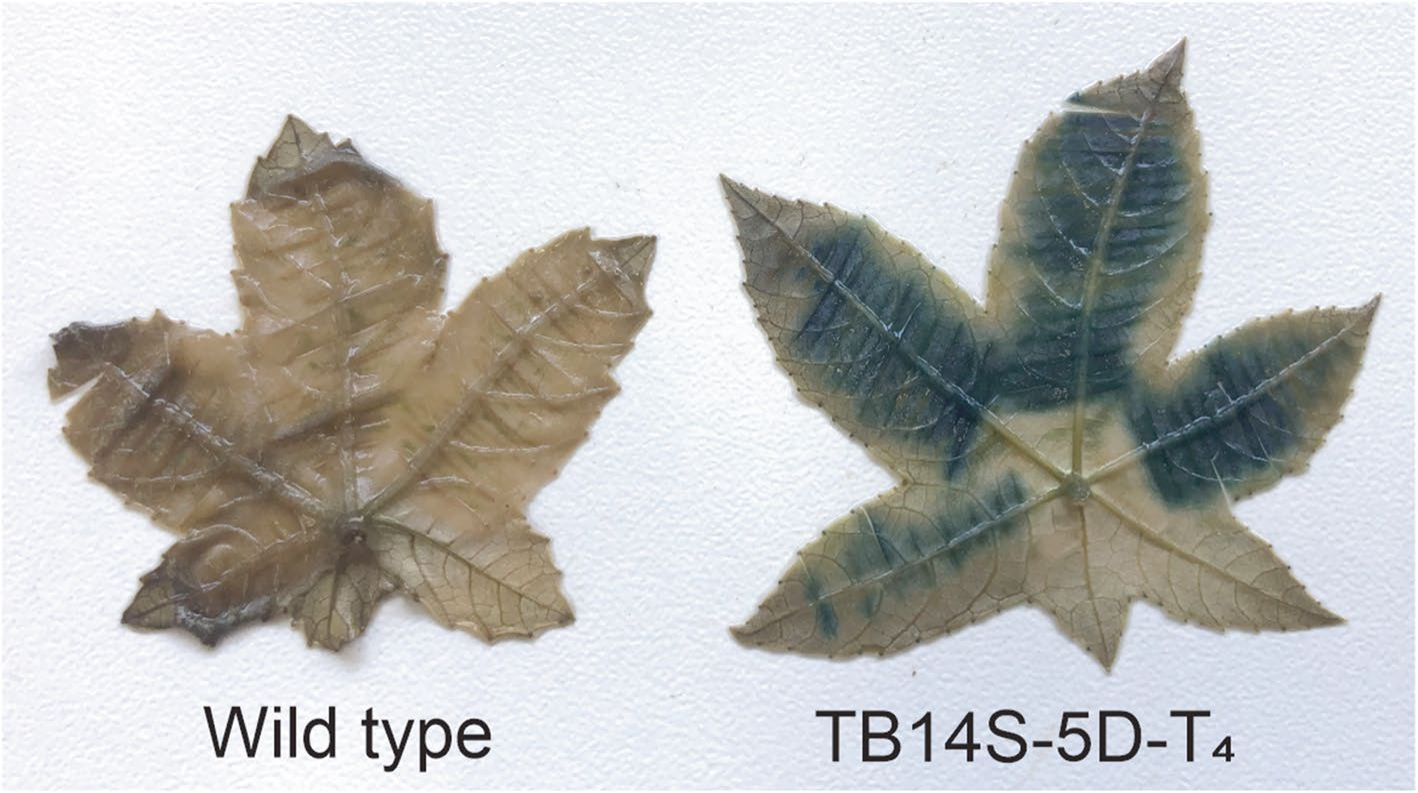
Expression of the *gus* gene in transgenic leaf from the 4th generation of event TB14S-5D (homozygous plants). Wild type is a leaf from the non-transgenic line. Leaves were cut to assist substrate penetration

No agglutination was observed in red blood cells (RBCs) incubated with transgenic seed protein extract (T_1_, T_2_, or T_3_ generations) (Fig. 3a), which shows the stably silenced RCA_120_. In contrast, hemagglutination was observed with purified RCA_120_ and WT proteins (Fig. 3a). ELISA was not able to detect ricin/RCA_120_ proteins from seeds of the TB14S-5D T_1_, T_2_, and T_3_ generations (Fig. 3b). Moreover, the TB14S-5D T_3_ and T_4_ generations (homozygous plants) showed strong *gus* expression in leaves (Fig. 4). Homozygosity was determined by testing 30 plants from the T_3_ generation for *gus* gene expression, which showed that all of them were positive (data not shown).

RNAi is an important tool in plant science and has been shown to be effective in silencing genes stably. In 2007, a transgenic common bean event resistant to bean golden mosaic virus (*BGMV*) using the RNAi strategy was developed [18]. After 17 years and over more than 24 generations, the transgenic event presents stable RNA silencing and resistance to *BGMV*, including in commercial areas (Thiago L.P.O Souza, Embrapa, Personal communication). In addition, RNAi has been an effective tool to generate transgenic crops resistant to insect pests, with the development of some commercial products [19].

## Conclusions

Collectively, our results demonstrated that event TB14S-5D is tolerant to the herbicide imazapyr and that ricin/RCA_120_ silencing is stable over three generations. This technology is a foundation for safer cultivation and industrial use of castor bean. Herbicide tolerance will help cultivation and harvesting of large areas. In addition, stable ricin/RCA_120_ silencing will allow castor cake to be used as an alternative animal foodstuff, due to its high nutritional value. Efforts are being made to evaluate event TB14S-5D under field conditions, as well as to introduce this trait to the breeding program and carry out biosafety analyses. This biotechnology will have a major impact on castor bean cultivation, allowing the production of ricinolein oil and protein sources for animal feeding in semi-arid and marginal areas, where the cultivation of other crops is difficult.

## Data Availability

The datasets used and/or analyzed during the current study are available from the corresponding author on reasonable request.

## Abbreviations

AHAS: Acetohydroxyacid synthase
GUS: β-Glucuronidase
IEC-6: Rat intestinal epithelial cell line
PBS: Phosphate-buffered saline
RBC: Red blood cells
RCA_120_: *R. communis* agglutinin
RIP: Ribosome-inactivating protein
RNAi: RNA interference
WT: Wild type
